# Effects of exogenous antioxidants on oxidative stress in pregnancy

**Published:** 2011-05-25

**Authors:** ML Staicu, A Mureşan, S Tache, R Moldovan

**Affiliations:** * Department of Medical Psychology, ‘Carol Davila’ University of Medicine and Pharmacy, BucharestRomania; ** Department of Psysiology, ‘Iuliu Haţieganu’ University of Medicine and Pharmacy, Cluj–NapocaRomania

**Keywords:** redox homeostasis, vitamin E, coenzyme Q_10_, gestation, primiparous, multiparous

## Abstract

**Objective**: The present study evaluated the effects on gestation, in terms of oxidative stress, of two antioxidant factors–vitamin E and coenzyme Q10–during pregnancy, with the purpose of applying the results in further human clinical practice.

**Methods**: For each aspect we have studied, we used three types of female rats of Wistar race (un–pregnant, primiparous, multiparous), divided in 10 rats/group. From the blood we have sampled, we have determined the oxidative stress (OS) markers: malondialdehyde (MDA) and carbonylated proteins (CP), but also the markers of the antioxidant defense: the hydrogen donor capacity of the plasma (HD) and the sulfhydryl groups (SH).

**Results**: Vitamin E administration determines significant decreases of MDA and significant increases of CP and HD at primiparous, and also significant increases of SH groups at multiparous. In the case of pregnant animals that received CoQ10 in antioxidant complexes, we have observed an increase of oxidative stress (OS)–MDA in primiparous and CP in multiparous.

**Conclusions**: In the case of Vitamin E, taking into account the benefits on redox homeostasis, the decrease of OS, the authors recommend vitamin E administration during pregnancy. However, because of the increase of the OS in the case of pregnant animals, the authors do not recommend the administration of CoQ_10_ in antioxidant complexes during pregnancy.

## Introduction

People are exposed to many carcinogens and the most important seem to be reactive oxygen species (ROS) and reactive nitrogen species (RNS). Formation of reactive oxygen species (ROS) and reactive nitrogen species (RNS) in the human body can cause oxidative damage to biological macromolecules such as DNA, lipids and proteins that may contribute to many diseases (cancer, cardiovascular and neurological diseases, etc). To counteract the prooxidant actions in the human body, an intricate network of antioxidants (AO) is operative in biological systems [1]. 

In a healthy body, there is a balance between ROS, RNS and AO. In normal pregnancies, the production of free radicals and lipoperoxidation increase towards the end of the pregnancy, as compared to unpregnant women. In the same time, the antioxidant capacity gradually increases during the pregnancy, leading to an oxidative balance that is maintained throughout the pregnancy [2]. On the other hand, a lack of balance between free radicals and antioxidants leads to **oxidative stress**.

The human diet provides a range of different compounds that possess antioxidant activities or have been suggested to scavenge ROS/RNS based on their structural properties. The most prominent representatives of dietary antioxidants are ascorbate (vitamin C), tocopherols (vitamin E), carotenoids and flavonoids [1]. Observational epidemiological studies clearly show a correlation between the increased consumption of food rich in antioxidants and a decreased risk of several oxidative stress related disease [3].  Protective effects of antioxidants have been found in animal studies [4], as well as in epidemiological studies [5] and in some small–scale intervention studies [6]. 

## Objective

The presence of ROS and RNS into the female genital tract and the data concerning the modifications of the oxidants/antioxidants balance during normal and pathologic pregnancy determined us to experimentally study the effects of some antioxidant factors–vitamin Eand coenzyme Q_10_ on pregnancy, with the purpose of applying the results in further clinical practice.  

The genetic resemblance between mice and humans and the reduced pregnancy duration (20–21 days) determined us to choose this species for experimental research on gestation. 

## Materials and methods

We used three types of female rats of Wistar race (10 weeks un–pregnant, 12 weeks primiparous, over 12 weeks multiparous) for each aspect we have studied, divided in 10 rats/group. From the blood we have sampled on the 21^st^– 22^nd^ gestation day, we have determined the oxidative stress markers: malondialdehyde (MDA) and carbonylated proteins (CP), but also the markers of the antioxidant defense:  the hydrogen donor capacity (HD) of the plasma and the sulfhydryl groups (SH). 

The results were compared to those we have obtained in control groups (unpreagnant, primiparous and multiparous females) that did not receive antioxidant factors. 

### Vitamin E administration

Vitamin E was administered under the form of injectable intramuscular solution 30 mg/ml; it was administrated in 4 mg/kg doses 

### Coenzyme Q_10_ administration 

Coenzyme Q_10_ (CoQ_10_ plus), containing 20 mg CoQ_10_, 1000 U.I. β carotene, 0,21 U.I. α–tocopherol and10 mg Selenium/cup, was administered in oil–like form by oral–pharynges tabulation, in doses of 0,42 mg CoQ_10_, 22 U.I. β carotene, 0,21 U.I. α–tocopherol and 0,20 mg Selenium/ kg/ animal. 

## Results

### Vitamin E administration 

Vitamin E administration determines significant decreases of MDA and significant increases of HD in unpregnant animals, compared to the control group. In the case of primiparous animals, we determined significant decreases of MDA and significant increases of CP and HD, while in the case of multiparous animals the administration determined insignificant modifications of the OS markers and significant increases of SH groups. 

As a result, Vitamin E enhances the antioxidant capacity, with significant decreases of CP and significant increases of SH, in multiparous and primiparous animals. 

**Figure 1:**
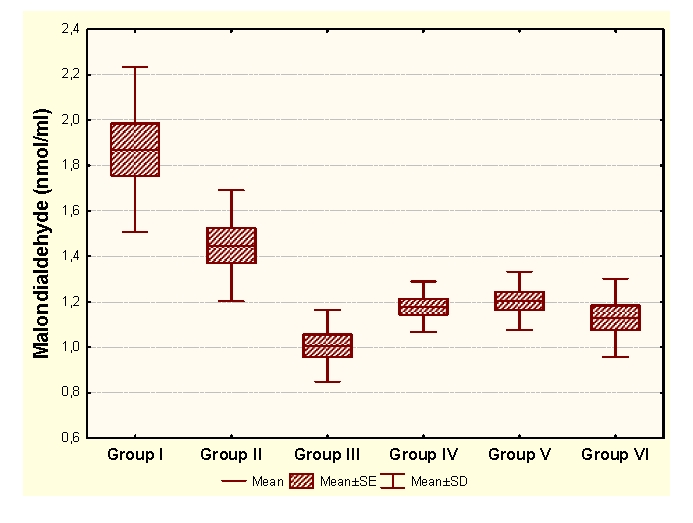
MDA at groups with/without Vitamin E administration

**Figure 2:**
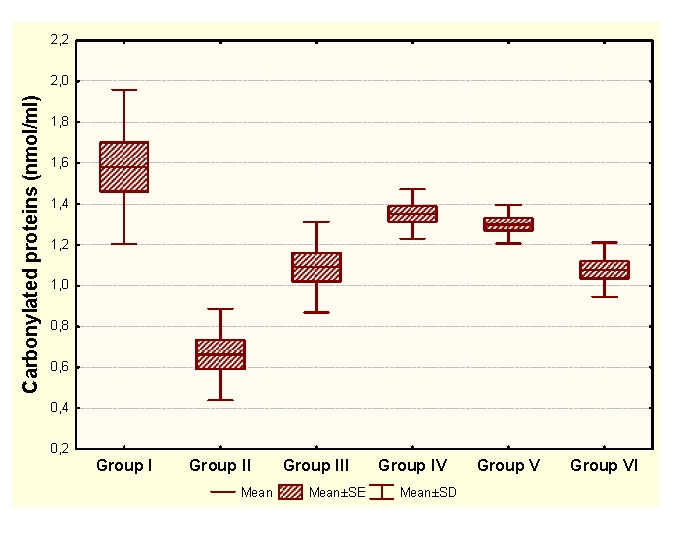
CP at groups with/without Vitamin E administration

**Figure 3:**
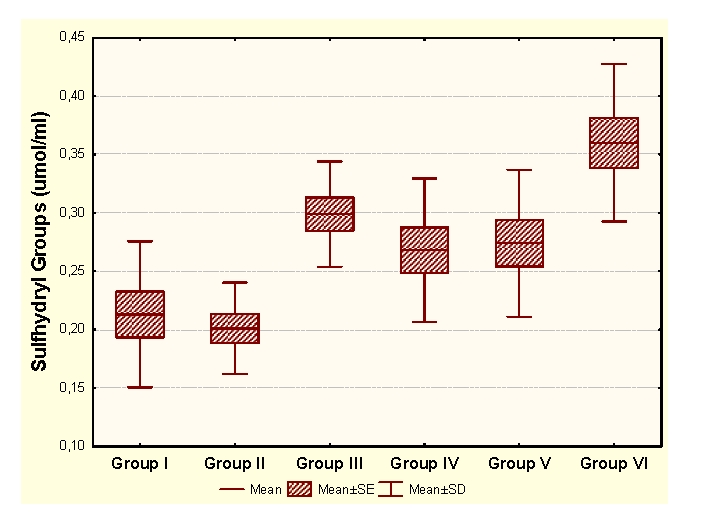
SH at groups with/without Vitamin E administration

**Figure 4:**
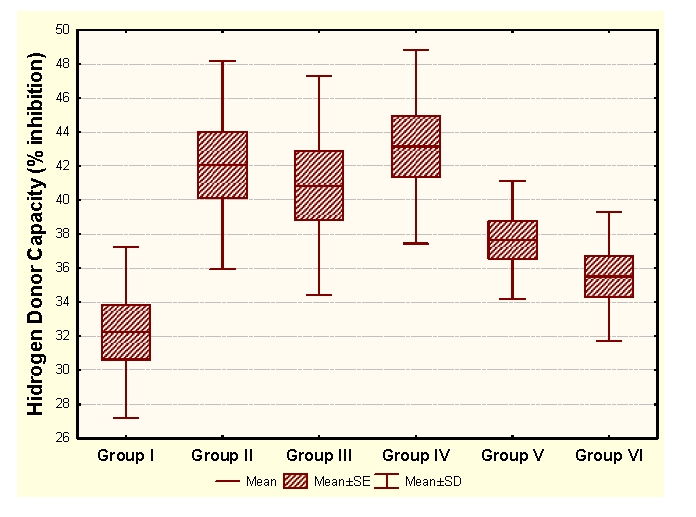
HD at groups with/without Vitamin E administration

Legend for figures Figure 1,Figure 2,Figure 3,Figure 4:

- Group I:unpregnant animals
- Group II: primiparous animals
- Group III: multiparous animals
- Group IV: unpregnant animals with vitamin E administration
- Group V: primiparous animals with vitamin E administration
- Group VI: multiparous animals with vitamin E administration

### CoQ10 administration 

The administration of an antioxidant complex determines significant increases of MDA and CP and significant increases of HD in unpregnant animals, compared to the control group. In the case of primiparous animals we determined insignificant modifications of MDA and HD and significant increases of CP and SH, and in the case of multiparous animals the administration of an antioxidant complex determines significant increases of MDA and insignificant modifications of CP and antioxidant capacity (HD and SH).

**Figure 5:**
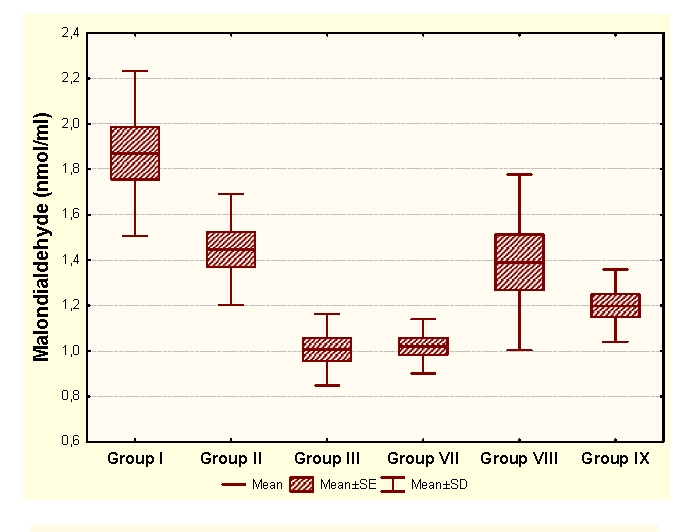
MDA at groups with/without CoQ10 administration

**Figure 6:**
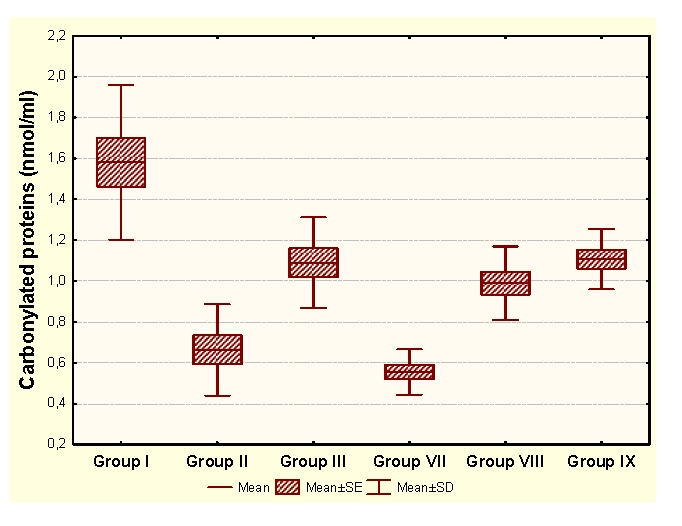
CP at groups with/without CoQ_10_ administration

**Figure 7:**
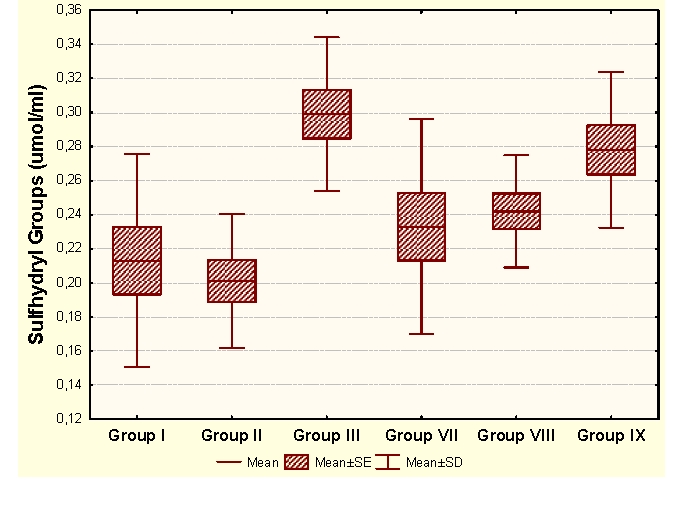
SH at groups with/without CoQ_10_ administration

**Figure 8:**
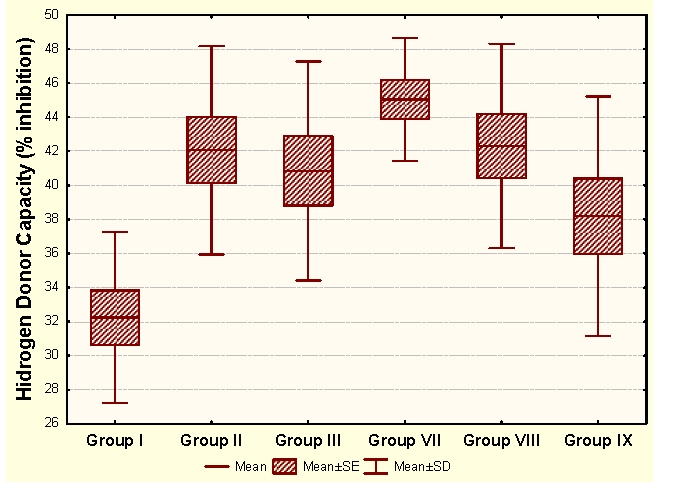
HD at groups with/without CoQ_10_ administration

Legend for figures 5,6,7,8:

- Group I:  unpregnant animals
- Group II: primiparous animals
- Group III: multiparous animals
- Group VII: unpregnant animals with CoQ_10_ administration
- Group VIII: primiparous animals with CoQ_10_ administration
- Group IX: multiparous animals with CoQ_10_ administration

## Discussions

Our research aimed to discover the consequences of antioxidant administration–Vitamin E and Coenzyme Q_10_ on pregnant animals, with the purpose of applying the results in further human clinical practice. 

Vitamin E is the best antioxidant found in membranes, inhibiting the peroxidation of lipids and the LDL oxidation; it stabilizes membranes, reduces the xathine oxidase and increases the antioxidant effect of Se. Its role during pregnancy has not made the object of many studies. 

Vitamin E is very important even in prenatal period, during the embryonic and fetal development of the conception product and continuing with the post–natal development of the child [7]. 

The recommended daily doses of Vitamin E is of 22–30 mg/day, quantity considered as sufficient to cover the nutrient need of an adult, including pregnant women. Vitamin E acts on the lipid membranes in order to prevent their oxidation and the forming of free radicals that could harm the cellular membranes [8]. 

The scientific literature reported that Vitamin E could play an important role in preventing: cardiovascular diseases and different types of cancer [8], chronic inflammations and neurological diseases [9], the bronchopulmonary dysplasia of the newborn [10], preeclampsia [11, 12, 13, 14]. Other data show that in high doses, Vitamin E does not reduce the incidence of preeclampsia [15]. 

Our results recommend Vitamin E administration during pregnancy, taking into account the benefic effects on redox homeostasis and the decrease of OS. In the case of the administration of AO complexes that contain Vitamin E as well the effects are not positive. 

CoQ_10_, vitamin–like complex, also known as vitamin Q_10_ or ubichinona–10 has the following roles: an essential redox transporter within the mitochondrial respiratory chain; antioxidant against the lipoperoxidation at the level of the internal mitochondrial membrane, the cellular membrane and for the low density lipids; favors the antioxidant action of Vitamin E; a central role in the energetic metabolism; increases the AO activity of vitamin C and β–carotene.

In case of preeclampsia, research has reported decreases of the CoQ_10_ plasmatic levels [16] and increases at the placenta level [17]. 

The maternal–fetal distribution of CoQ_10_ can increase the risk of oxidative lesions for the newborn [18]. The placental increase of CoQ_10_ in the case of Hellp syndrome, a severe complication of preeclampsia, can be another compensating mechanism in the case of OS increase [19]. The presence of CoQ_10_ in high concentrations in the maternal milk can be directly correlated with its AO capacity in different lactation stages, in the case of preterm or normal deliveries [20]. 

Other data suggest that a rich AO diet–CoQ_10_  and Vitamin E before and during pregnancy does not decrease the incidence of malformations inducted by the administration of phenytoin in female rats [21]. 

Our results show the importance of an O/AO balance control and they agree with the literature data concerning the negative effects of CoQ_10_  administration during pregnancy. Our study observed the increase of OS in pregnant animals that received CoQ_10_. In conclusion, the administration of CoQ_10_  in antioxidant complexes is not recommended during pregnancy. The negative effect also appears in the case of AO complexes composed of CoQ_10_, Se, α–tocopherol and β–carotene.

## Conclusions

Our research brings experimental proofs regarding the effect on redox homeostasis of some antioxidant factors during pregnancy (Vitamin E and CoQ10 administration); our study agrees with the scientific literature that determined the consequences of these factors on human pregnancy. 

In the case of Vitamin E, taking into account the positive effects on redox homeostasis, with the decrease of OS, the authors recommend the Vitamin E administration during pregnancy. On the other hand, because of the increase of the OS in the case of pregnant animals, the authors do not recommend the administration of CoQ_10_ in antioxidant complexes during pregnancy.  
